# Supplementary material for: Deciphering Microbial Composition in Patients with Inflammatory Bowel Disease: Implications for Therapeutic Response to Biologic Agents
Source: Microorganisms. 2024 Jun 21;12(7):1260. doi: 10.3390/microorganisms12071260 (PMC11278628; doi:10.3390/microorganisms12071260)

Supplementary File 1

1.1 – Sequencing and alignment statistics.

Per- sample Sequencing statistics. First column: disease status; second column: Sample identifier; columns 3 – 8: absolute counts and percentages for surviving reads after QIIME2 DADA2 implementation.

	Sample ID	#reads	#filtered	%filtered	#denoised	#merged	% input merged	# non-chimeric	% non-chimeric
Healthy control	10C	115538	75888	65.68	72743	60144	52.06	32116	27.8
Healthy control	11C	333911	223628	66.97	218224	195657	58.6	135087	40.46
Healthy control	12C	161464	113144	70.07	112019	105980	65.64	68036	42.14
Healthy control	1C	221871	130960	59.03	128773	117380	52.9	66234	29.85
Healthy control	2C	409927	289527	70.63	286851	275230	67.14	222845	54.36
Healthy control	4C	402377	295734	73.5	288931	260108	64.64	182688	45.4
Healthy control	5C	292050	180823	61.92	176199	152952	52.37	80789	27.66
Healthy control	7C	324335	211693	65.27	207732	186987	57.65	107853	33.25
Healthy control	8C	541443	383477	70.82	375828	338007	62.43	213912	39.51
Healthy control	CON652SGR	193330	131073	67.8	124321	98850	51.13	59422	30.74
Healthy control	CON653SGR	218667	153939	70.4	148707	125196	57.25	79173	36.21
Healthy control	CON658SGR	183060	135921	74.25	129535	107086	58.5	57088	31.19
Healthy control	CON659SGR	238868	188881	79.07	182876	158889	66.52	71404	29.89
Healthy control	CON660SGR	159146	116230	73.03	112897	95740	60.16	49645	31.19
Healthy control	CON661SGR	208649	134210	64.32	130170	111609	53.49	68905	33.02
Healthy control	CON662SGR	109757	84082	76.61	80667	65030	59.25	38775	35.33
Healthy control	CON663SGR	268645	192054	71.49	187117	165110	61.46	82185	30.59
Healthy control	CON664SGR	122688	76226	62.13	74364	66080	53.86	45568	37.14
Healthy control	CON665SGR	172435	130346	75.59	126724	109669	63.6	63649	36.91
Healthy control	CON666SGR	167347	115609	69.08	113689	103966	62.13	65551	39.17
Healthy control	CON667SGR	124097	84072	67.75	82038	71992	58.01	42584	34.32
Healthy control	CON668SGR	246404	182728	74.16	180671	170295	69.11	106873	43.37
Healthy control	CON669SGR	202119	160008	79.17	157975	148679	73.56	101573	50.25
Healthy control	CON670SGR	260862	199447	76.46	196867	185786	71.22	105921	40.6
Healthy control	CON671SGR-	302717	205846	68	202615	187008	61.78	133045	43.95

Healthy control	D01SGR	295998	218843	73.93	213300	190939	64.51	119438	40.35
Healthy control	D02SGR	145683	102347	70.25	99942	87796	60.27	58599	40.22
Healthy control	D03SGR	197762	124173	62.79	119344	97887	49.5	54368	27.49
Healthy control	D04SGR	122531	90207	73.62	87607	76014	62.04	43846	35.78
CD_baseline	CD1728SGR_baseline	211392	167033	79.02	166255	162914	77.07	129580	61.3
CD_52_weeks	CD1728SGR_52-weeks	242459	186906	77.09	185839	181987	75.06	138188	56.99
CD_baseline	CD216SGR_baseline	309011	212287	68.7	209896	197342	63.86	88216	28.55
CD_baseline	CD2207SGR_baseline	194623	150143	77.15	148752	141946	72.93	90857	46.68
CD_baseline	CD3290SGR_baseline	173937	123622	71.07	121830	112194	64.5	64467	37.06
CD_14_weeks	CD3290SGR_14-weeks	137846	95004	68.92	93336	84611	61.38	57590	41.78
CD_52_weeks	CD3290SGR_52-weeks	228481	154128	67.46	151358	136398	59.7	71519	31.3
CD_baseline	CD5292SGR_baseline	147709	106280	71.95	105061	99022	67.04	55678	37.69
CD_baseline	CD5310SGR_baseline	175356	128091	73.05	126941	122506	69.86	78159	44.57
CD_baseline	CD5318SGR_baseline	173010	114551	66.21	112484	102793	59.41	59858	34.6
CD_baseline	CD5320SGR_baseline	166520	128709	77.29	127030	119233	71.6	72697	43.66
CD_baseline	CD5321SGR_baseline	221369	156466	70.68	154705	148010	66.86	112380	50.77
CD_baseline	CD5333SGR_baseline	171028	117394	68.64	116296	110954	64.87	71645	41.89
CD_baseline	CD5339SGR_baseline	149680	101343	67.71	100284	96256	64.31	71329	47.65
CD_baseline	CD5341SGR_baseline	253005	136942	54.13	135951	131964	52.16	108875	43.03
CD_14_weeks	CD5341SGR_14-weeks	161576	114225	70.69	112952	106265	65.77	77480	47.95
CD_52_weeks	CD5341SGR_52-weeks	120039	74687	62.22	73694	69383	57.8	54299	45.23
CD_baseline	CD5342SGR_baseline	185393	128665	69.4	126851	116093	62.62	52100	28.1
CD_baseline	CD5355SGR_baseline	196097	133433	68.04	133014	131633	67.13	117179	59.76
CD_baseline	CD5377SGR_baseline	592818	379980	64.1	374726	338307	57.07	172685	29.13
CD_14_weeks	CD5377SGR_14-weeks	203903	123768	60.7	121752	110071	53.98	76707	37.62
CD_52_weeks	CD5377SGR_52-weeks	253270	170468	67.31	168500	157493	62.18	125547	49.57
CD_baseline	CD5390SGR_baseline	213204	162261	76.11	160340	149567	70.15	92691	43.48
CD_baseline	CD5391SGR_baseline	158600	123638	77.96	122445	118196	74.52	79837	50.34
CD_baseline	CD5419SGR_baseline	167959	86175	51.31	85227	76809	45.73	54862	32.66
CD_baseline	CD5426SGR_baseline	175735	118008	67.15	115839	103649	58.98	48720	27.72
CD_baseline	CD5445SGR_baseline	142879	107421	75.18	106321	102207	71.53	62367	43.65
UC_baseline	UC3314SGR_baseline	252722	187623	74.24	184298	167736	66.37	92243	36.5
UC_14_weeks	UC3314SGR_14-weeks	380083	281362	74.03	276878	248106	65.28	113963	29.98
UC_52_weeks	UC3314SGR_52-weeks	246040	178126	72.4	175757	160279	65.14	104441	42.45
UC_baseline	UC3366SGR_baseline	221053	146790	66.4	142957	123101	55.69	52766	23.87
UC_52_weeks	UC3366SGR_52-weeks	168311	123963	73.65	121137	106840	63.48	43360	25.76
UC_baseline	UC3472SGR_baseline	101045	59639	59.02	57398	47354	46.86	26571	26.3
UC_14_weeks	UC3472SGR_14-weeks	267912	190242	71.01	187031	173658	64.82	114285	42.66
UC_52_weeks	UC3472SGR_52-weeks	145332	100614	69.23	98492	88289	60.75	61450	42.28
UC_baseline	UC3825SGR_baseline	206641	134442	65.06	133674	117776	57	70446	34.09

UC_baseline	UC3970SGR_baseline	204115	151834	74.39	149745	140620	68.89	84002	41.15
UC_baseline	UC4310SGR_baseline	223107	145532	65.23	143914	131790	59.07	61201	27.43
UC_14_weeks	UC4310SGR_14-weeks	142926	76464	53.5	75458	68205	47.72	42596	29.8
UC_52_weeks	UC4310SGR_52-weeks	152114	113417	74.56	111448	100577	66.12	52327	34.4
UC_baseline	UC4418SGR_baseline	194438	141381	72.71	139983	132306	68.05	84948	43.69
UC_baseline	UC5011SGR_baseline	218635	168950	77.27	163482	135965	62.19	56834	25.99
UC_baseline	UC5195SGR_baseline	109203	80264	73.5	77715	66089	60.52	40018	36.65
UC_14_weeks	UC5195SGR_14-weeks	178225	124659	69.94	121169	104927	58.87	57144	32.06
UC_52_weeks	UC5195SGR_52-weeks	109652	82610	75.34	79937	68150	62.15	37229	33.95
UC_baseline	UC5245SGR_baseline	99615	73139	73.42	71242	63080	63.32	33857	33.99
UC_baseline	UC5300SGR_baseline	218199	167590	76.81	164301	146133	66.97	55667	25.51
UC_baseline	UC5311SGR_baseline	333802	236226	70.77	233229	219744	65.83	155799	46.67
UC_14_weeks	UC5311SGR_14-weeks	233477	160089	68.57	156714	139344	59.68	76143	32.61
UC_52_weeks	UC5311SGR_52-weeks	171640	122789	71.54	119702	106030	61.77	58449	34.05
UC_baseline	UC5322SGR_baseline	146821	103511	70.5	102418	96953	66.03	61686	42.01
UC_14_weeks	UC5322SGR_14-weeks	182450	130324	71.43	129125	124315	68.14	71236	39.04
UC_52_weeks	UC5322SGR_52-weeks	431486	311089	72.1	308664	296443	68.7	112746	26.13
UC_baseline	UC5325SGR_baseline	228580	173794	76.03	171969	163607	71.58	118074	51.66
UC_14_weeks	UC5325SGR_14-weeks	153805	106028	68.94	103415	91889	59.74	52593	34.19
UC_52_weeks	UC5325SGR_52-weeks	207468	134849	65	132376	119864	57.77	59958	28.9
UC_baseline	UC5381SGR_baseline	220812	162863	73.76	161270	153450	69.49	115509	52.31
UC_baseline	UC5407SGR_baseline	228965	181627	79.33	179038	165934	72.47	72899	31.84
UC_52_weeks	UC5407SGR_52-weeks	820503	645086	78.62	634335	573298	69.87	251305	30.63
UC_baseline	UC5436SGR_baseline	544120	403107	74.08	395014	349156	64.17	170330	31.3
UC_52_weeks	UC5436SGR_52-weeks	165224	118621	71.79	114390	97227	58.85	52954	32.05
UC_baseline	UC5437SGR_baseline	163828	120960	73.83	118115	100923	61.6	41535	25.35
UC_52_weeks	UC5437SGR_52-weeks	170003	125464	73.8	123015	109276	64.28	55148	32.44
UC_baseline	UC5486SGR_baseline	198047	144317	72.87	141742	126337	63.79	72449	36.58
UC_14_weeks	UC5486SGR_14-weeks	171514	124866	72.8	123235	114645	66.84	75884	44.24
UC_52_weeks	UC5486SGR_52-weeks	232517	167804	72.17	165408	149575	64.33	67966	29.23

1.2 - Feature distribution per sample ("per sample" column) and global feature distribution ("per feature").

The number of high-quality sequences counts ranges from 26571 to 251305, while feature abundance ranges from 1 to 394808 counts.

	Per sample	*Per feature*
Min. frequency	26571	1
1st quartile	55672	3
Median frequency	71329	6
3rd quartile	105181	62
Max. frequency	251305	394808
Mean frequency	82849	841

1.2.1 Numberof patients analyzed in each group and subgroup throughout the study.

	*Case-Control study	*Biologic Therapies 14 Weeks		Biologic Therapies 52 Weeks		Vedolizumab 14 Weeks		Vedolizumab 52 Weeks		Anti-TNF 14 Weeks		Anti-TNF 52 Weeks	
	Number of Subjects	Responders	Non Responders	Responders	Non Responders	Responders	Non Responders	Responders	Non Responders	Responders	Non Responders	Responders	Non Responders
IBD	39	22	16	18	20	14	11	11	14	8	5	7	6
CD	20	12	7	9	10	7	5	4	8	5	2	5	2
UC	19	10	9	9	10	7	6	7	6	3	3	2	4
HC	20	-	-	-	-	-	-	-	-	-	-	-	-

*The CD patient who received Ustekinumab was included only in the case-control study; for the response to biologic therapies the total of the IBD patients were 38.

1.3 - Alpha diversity analysis.

We investigated Alpha diversity by calculating four different measures: Pielou's evenness (1.4.1), Faith's Phylogenetic Distance (1.4.2), Number of Observed Features (1.4.3), and Shannon's entropy (1.4.4). For each index, the following outcomes are provided: boxplot for group-specific index distribution (x-axis: "HC", "IBD", "CD", and "UC"; y-axis: alpha-diversity index); Kruskal-Wallis test results (H statistics; p-value) for the global comparison of group-specific distribution; Kruskal-Wallis test results for pairwise group comparisons. Boxplot 1.4.2 is also reported in the main text, Figure 1. Analyses were performed through the "QIIME2 Diversity" module, using "**20000**" as the cut-off for reads sampling depth (see main text for further details).

Pielou's evenness

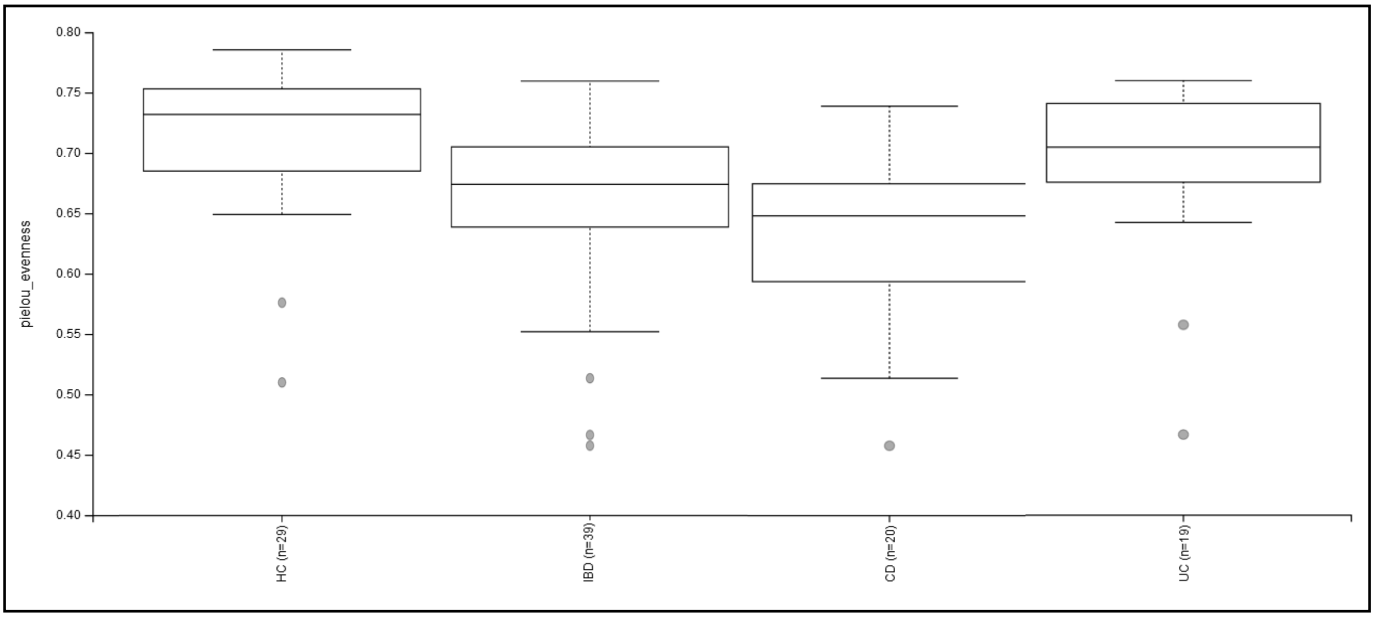

Kruskal-Wallis test (pairwise):

Group 1	Group 2	H	p-value	q-value
**HC (n=29)**	**IBD (n=39)**	11.09	0.000870	0.001909
	**CD (n=20)**	17.90	0.000023	0.000140
	**UC (n=19)**	1.63	0.202141	0.202141
**CD (n=20)**	**UC (n=19)**	10.08	0.001498	0.002996

Faith's Phylogenetic Distance. The figure is also reported in the Main Text, as Figure 1

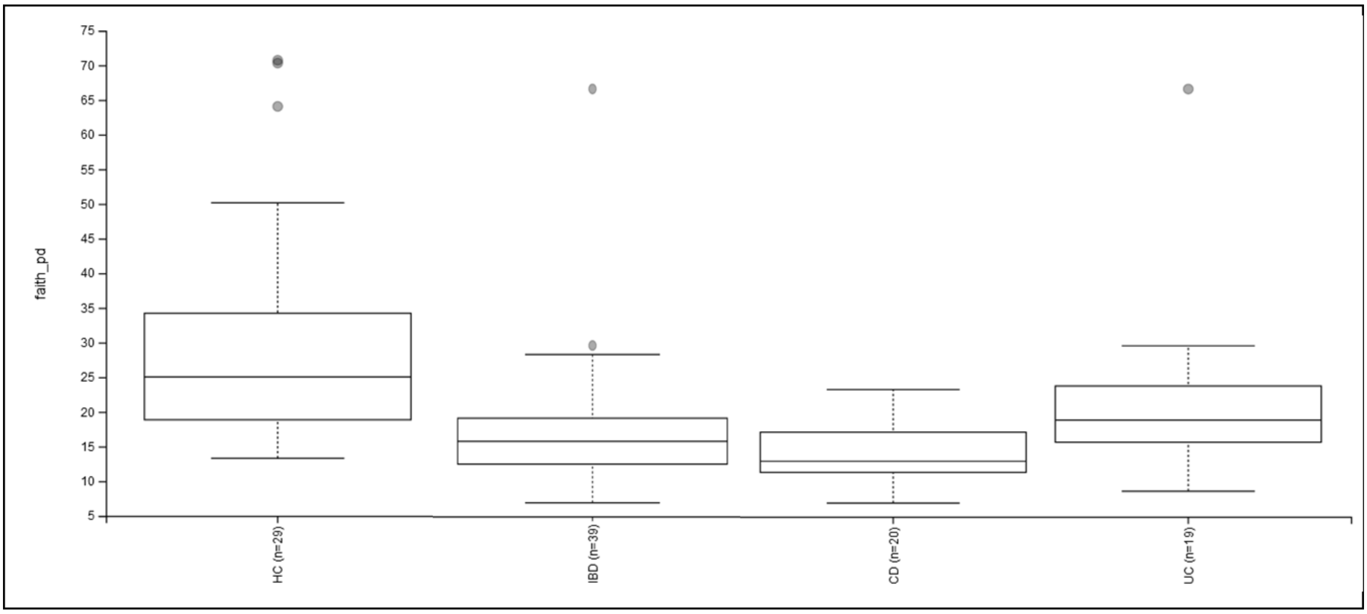

Kruskal-Wallis test (pairwise):

Group 1	Group 2	H	p-value	q-value
**HC (n=29)**	**IBD (n=39)**	20.655	0.000006	0.000017
	**CD (n=20)**	25.450	4.540139e-07	0.000003
	**UC (n=19)**	6.241	0.0125	0.014977
**CD (n=20)**	**UC (n=19)**	7.428	0.0064	0.012842

Number of Observed Features

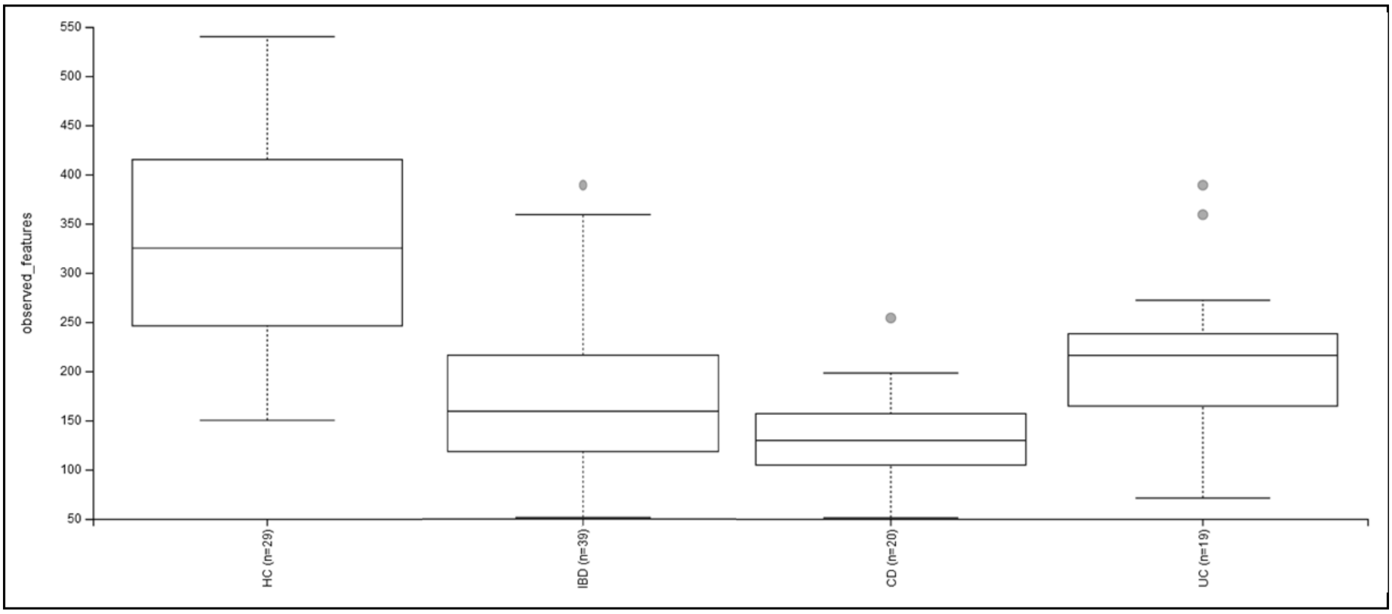

Kruskal-Wallis test (pairwise):

Group 1	Group 2	H	p-value	q-value
**HC (n=29)**	**IBD (n=39)**	29.707213	5.024750e-08	1.507425e-07
	**CD (n=20)**	30.621881	3.135378e-08	1.881227e-07
	**UC (n=19)**	12.474646	0.00041	0.000665
**CD (n=20)**	**UC (n=19)**	12.340522	0.00044	0.000665

Shannon entropy

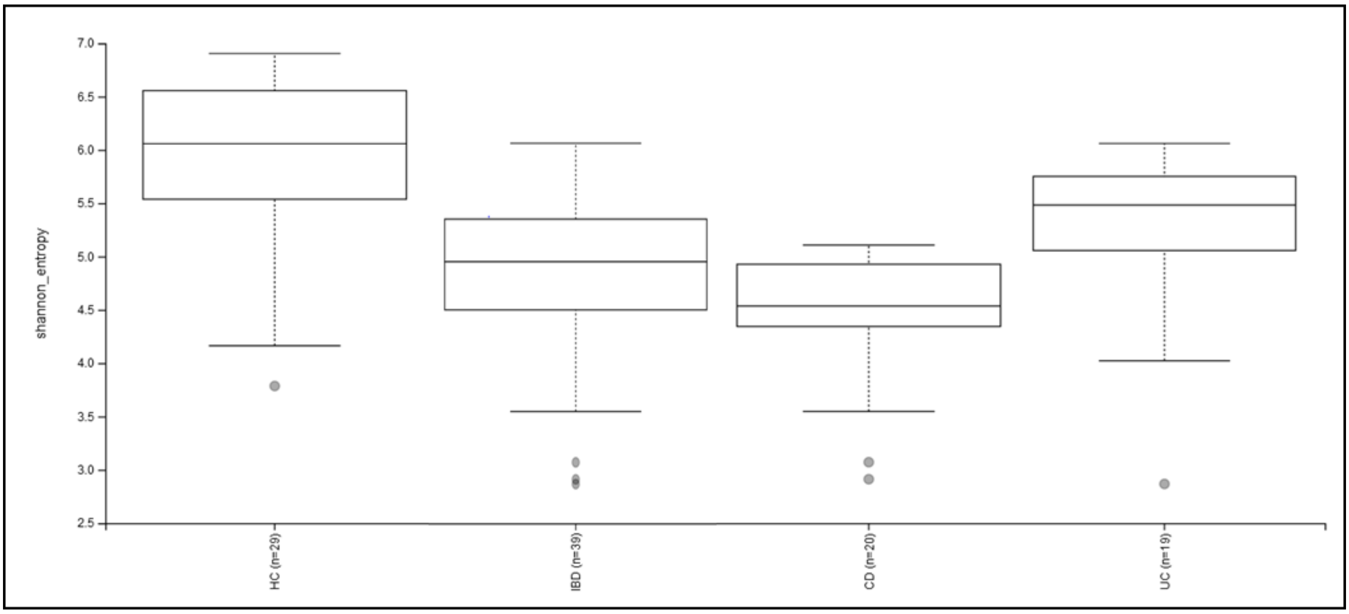

Kruskal-Wallis test (pairwise):

Group 1	Group 2	H	p-value	q-value
**HC (n=29)**	**IBD (n=39)**	24.541614	7.272216e-07	0.000002
	**CD (n=20)**	26.486483	2.653886e-07	0.000002
	**UC (n=19)**	9.539131	0.0020	0.003017
**CD (n=20)**	**UC (n=19)**	15.695526	7.439993e-05	0.000149

1.4 - Beta diversity analysis.

Diversity between the two groups has been evaluated by considering four popular dissimilarity/distance methods: Bray-Curtis (1.5.1), Jaccard (1.5.2), Unweighted and Weighted Unifrac (1.5.3 and 1.5.4). For each method, the following outcomes are provided: a screenshot of a three-dimensional Principal Coordinate Analysis ("3D-PCoA") plot; and a table summarizing PERMANOVA test results for pairwise group comparisons. PCoA plots have been obtained by using the Emperor web-application within the QIIME2 website (https://view.qiime2.org/); blue dots indicate "HC" samples, red "CD", and green "UC". Furthermore, each table shows details on comparing groups and their size, number of permutations, pseudo-F statistics, p-values, and Benjamini-Hochberg adjusted p-value. PERMANOVA verifies the hypothesis that distances among samples with one class group differ from distances of these samples from samples of other groups. PCoA plot obtained by using Bray-Curtis distance is also reported in the Main Text, as Figure 2.

Bray-Curtis

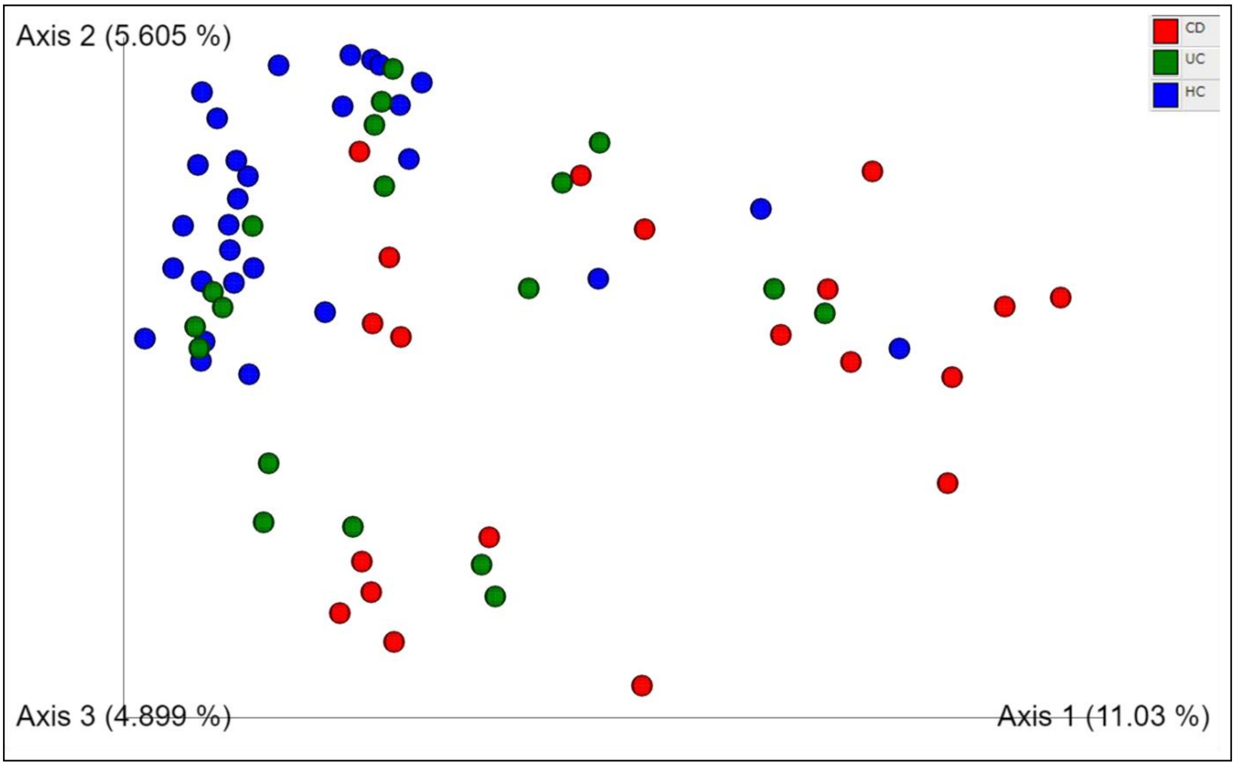

Group 1	Group 2	Sample size	Permutations	pseudo-F	p-value	q-value
**HC**	**IBD**	68	9999	3.441030	0.0001	0.00015
	**CD**	49	9999	4.164666	0.0001	0.00020
	**UC**	48	9999	2.324556	0.0001	0.00020
**CD**	**UC**	39	9999	2.357689	0.0002	0.00030

Jaccard

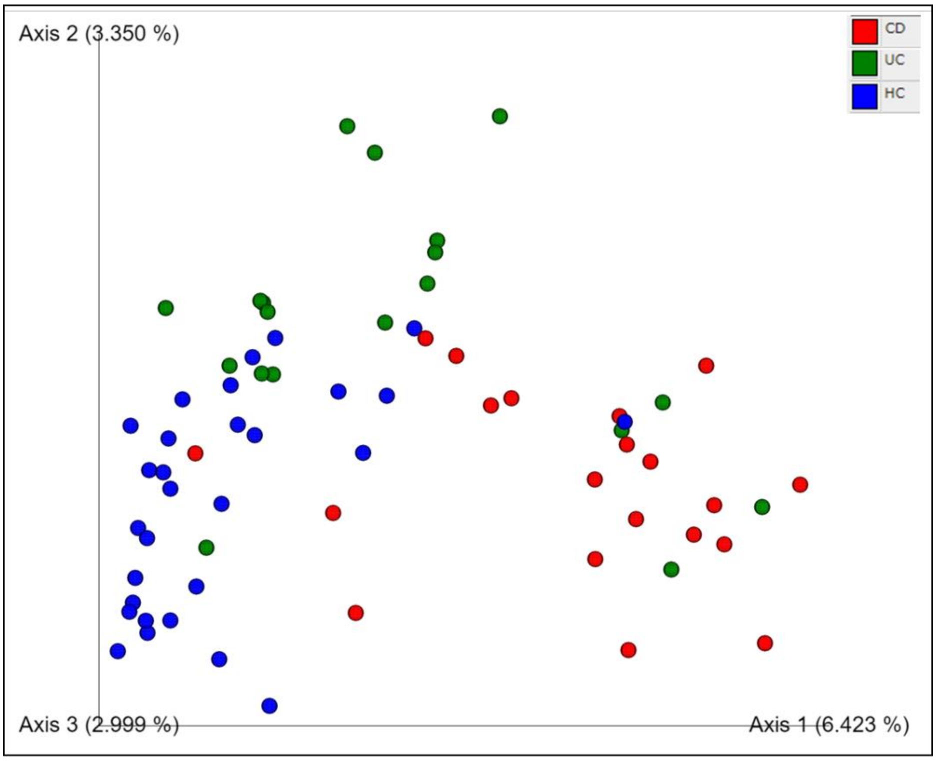

Group 1	Group 2	Sample size	Permutations	pseudo-F	p-value	q-value
**HC**	**IBD**	68	9999	2.866650	0.0001	0.00015
	**CD**	49	9999	3.302674	0.0001	0.00020
	**UC**	48	9999	1.942408	0.0001	0.00020
**CD**	**UC**	39	9999	1.805797	0.0002	0.00024

Unweighted Unifrac

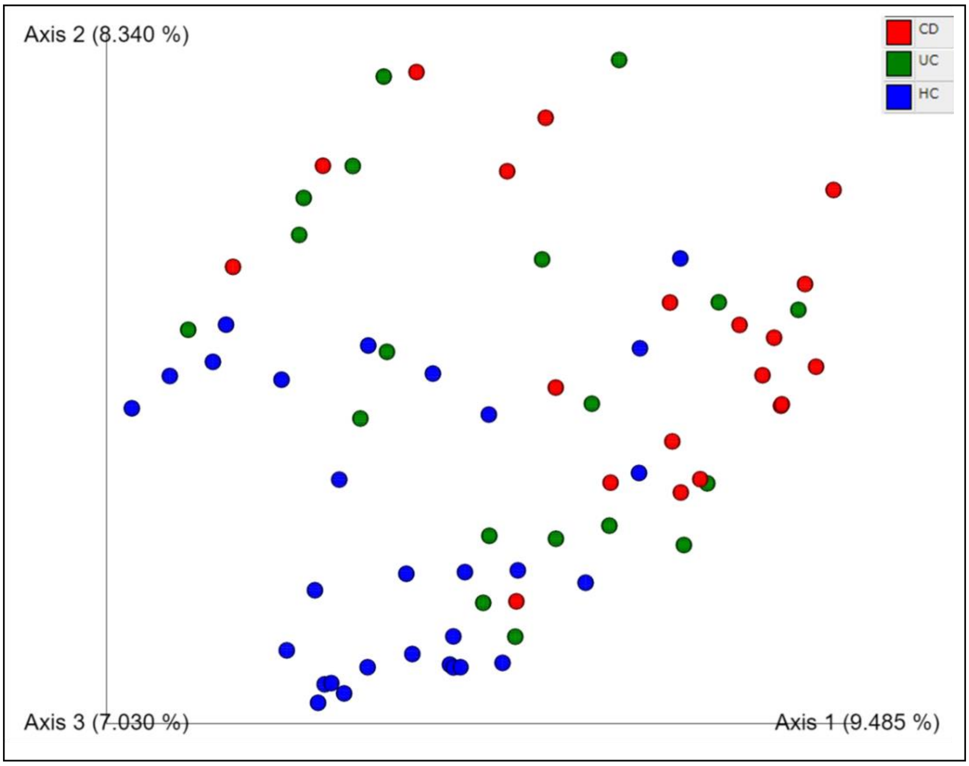

Group 1	Group 2	Sample size	Permutations	pseudo-F	p-value	q-value
**HC**	**IBD**	68	9999	4.366697	0.0001	0.00015
	**CD**	49	9999	4.578665	0.0001	0.00020
	**UC**	48	9999	2.646917	0.0001	0.00020
**CD**	**UC**	39	9999	1.747220	0.0056	0.00672

Weighted Unifrac

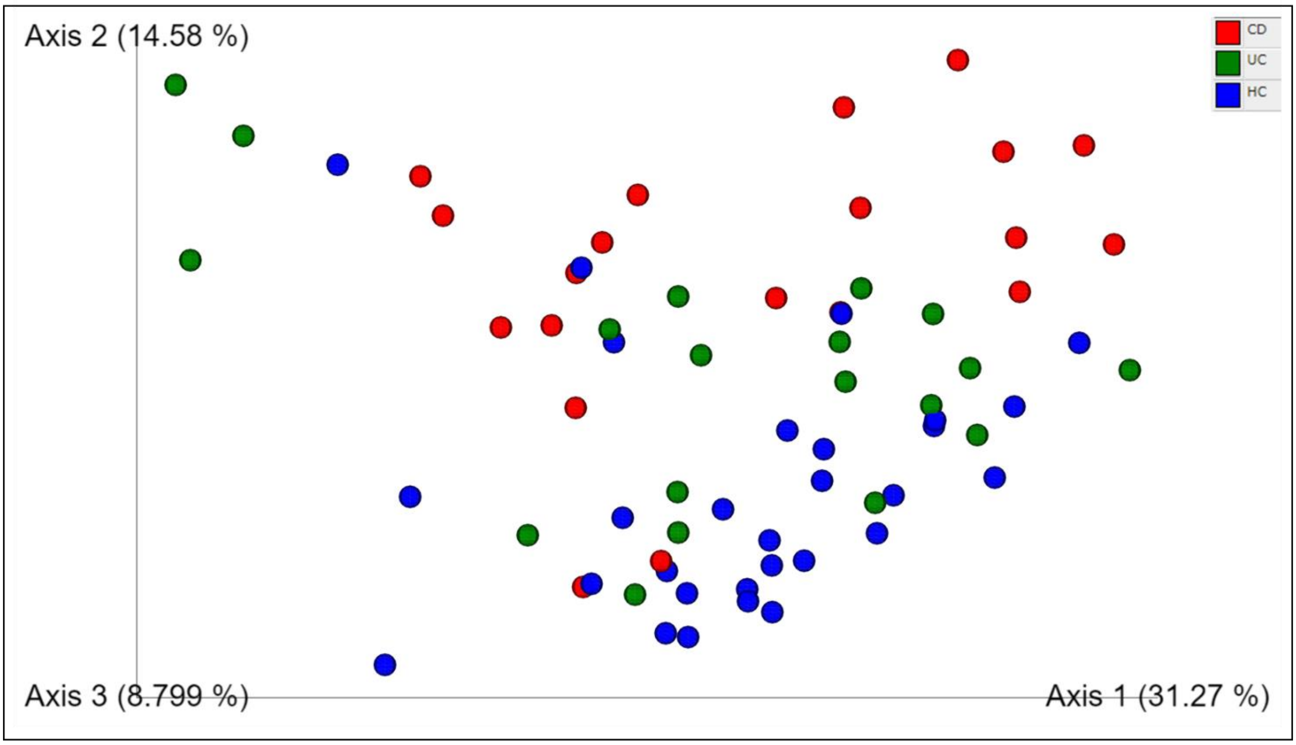

Group 1	Group 2	Sample size	Permutations	pseudo-F	p-value	q-value
**HC**	**IBD**	68	9999	4.710726	0.0006	0.00180
	**CD**	49	9999	5.716614	0.0002	0.00120
	**UC**	48	9999	2.567463	0.0248	0.04960
**CD**	**UC**	39	9999	1.448809	0.1748	0.26220

1.5 – ANCOM

QIIME2 ANCOM module was used to infer microbial genera that are differentially abundant across sample groups. Rare features and mitochondrial/plastid sequences were removed from the feature table (see Methods). Thus, features were collapsed according to QIIME2 taxonomical classification (based on GreenGenes resource) into "genera-collapsed" ("level 6") features, for a total of 108 taxa. ANCOM Volcano Plot and statistical results table are provided.

Significant features are placed on the top-right corner of the Volcano Plot; the first table shows significant features together with the corresponding W statistics, i.e., the number of sub-hypotheses that have passed for a certain feature (ANCOM compares pairs of feature relative abundances). The second table shows the percentile abundance of relevant features across the sample groups. For example, a value of 100 at 50% percentile indicates that the detected feature has a maximum sequence count of 100 in the 50% of samples of an investigated group.

1.5.1 - Results for genus-collapsed features (HC vs IBD)

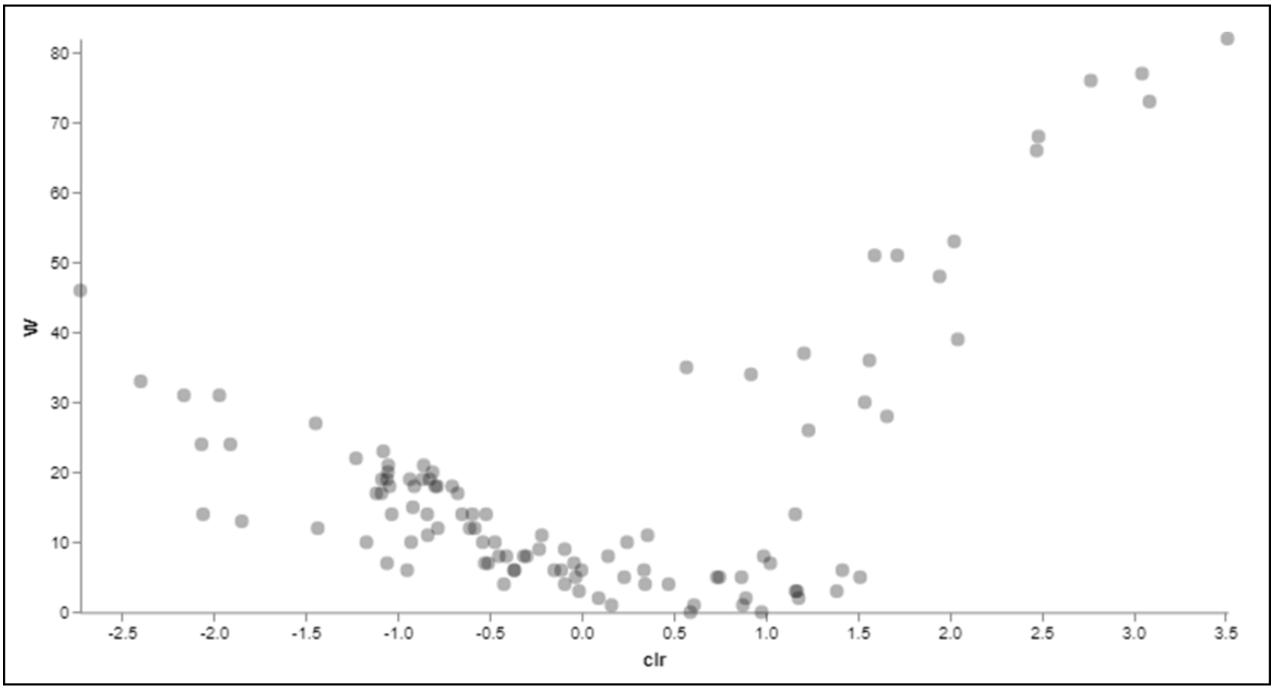

Features

ANCOM statistical results<br>Feature	**W**
k__Bacteria;p__Firmicutes;c__Clostridia;o__Clostridiales;f__Ruminococcaceae;g__Gemmiger	82
k__Bacteria;p__Firmicutes;c__Clostridia;o__Clostridiales;f__;g__	77
k__Bacteria;p__Bacteroidetes;c__Bacteroidia;o__Bacteroidales;f__Rikenellaceae;g__	76
k__Bacteria;p__Bacteroidetes;c__Bacteroidia;o__Bacteroidales;f__Rikenellaceae;g__Alistipes	73

Percentile<br>Group	**0.0**<br>**HC**	**25.0**<br>**HC**	**50.0**<br>**HC**	**75.0**<br>**HC**	**100.0**<br>**HC**	**0.0**<br>**IBD**	**25.0**<br>**IBD**	**50.0**<br>**IBD**	**75.0**<br>**IBD**	**100.0**<br>**IBD**
k__Bacteria;p__Firmicutes;c__Clostridia;<br>o__Clostridiales;f__Ruminococcaceae;g__Gemmiger	1	671	1081	2458	15486	1	1	1	654	4725
k__Bacteria;p__Firmicutes;c__Clostridia;<br>o__Clostridiales; f__;g__	1	13	143	362	8760	1	1	1	1	1441
k__Bacteria;p__Bacteroidetes;c__Bacteroidia;<br>o__Bacteroidales;f__Rikenellaceae;g__	1	103	345	1085	6953	1	1	1	54.5	2443
k__Bacteria;p__Bacteroidetes;c__Bacteroidia;<br>o__Bacteroidales;f__Rikenellaceae;g__Alistipes	1	494	1312	3476	29917	1	1	11	521	2992

1.5.2 - Results for genus-collapsed features (HC vs CD vs UC)

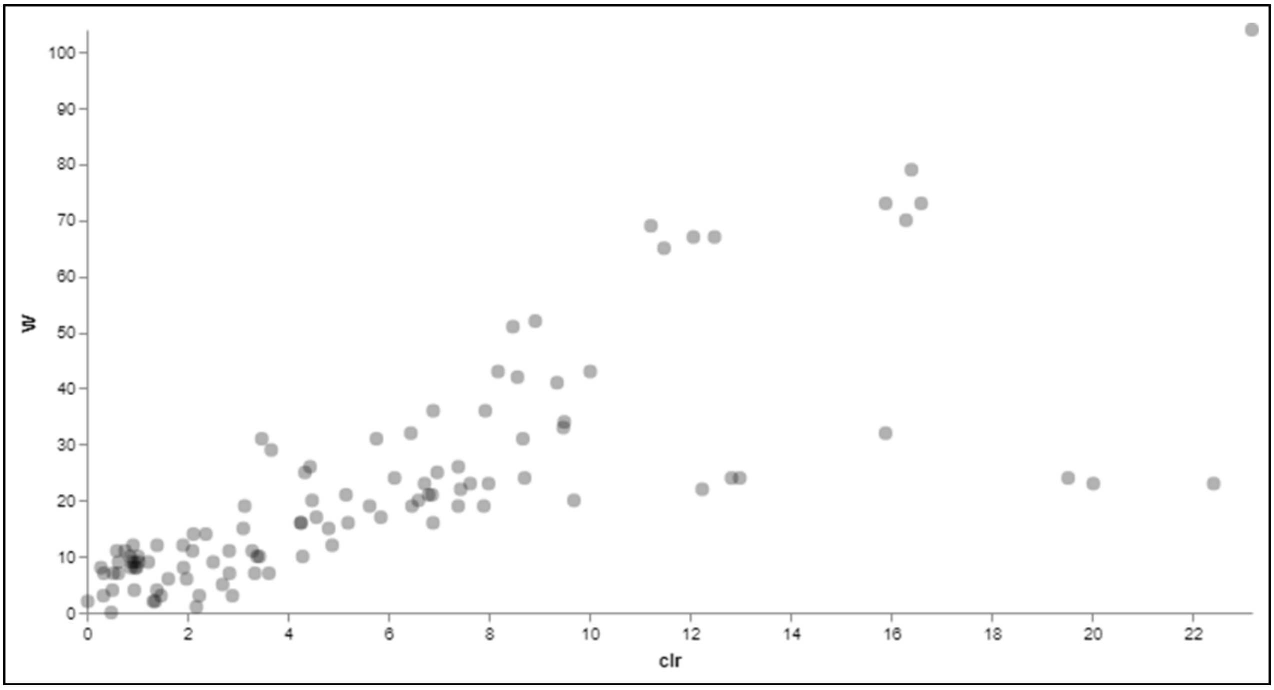

Feature	W
k__Bacteria;p__Firmicutes;c__Clostridia;o__Clostridiales;f__Lachnospiraceae;g__Clostridium	104

Feature	Percentile	HC	CD	UC
k__Bacteria;p__Firmicutes;c__Clostridia;o__Clostridiales;f__Lachnospiraceae;g__Clostridium	0.0	1	1	1
	25.0	23	326.25	1
	50.0	88	2101.5	35
	75.0	286	7368.25	172
	100.0	8917	34333	2001

1.5.3 - Results of the microbial ecosystem analysis according to the Lloyd-Price test

The degree of dysbiosis among all individuals with IBD at baseline and HC samples was assessed using the Lloyd-Price test (panel A, i.e., density plot for the Bray-Curtis pairwise distance for the two main sample groups). Panel B presents a box plot illustrating the distribution of the degree of dysbiosis in patients who responded or did not respond to Vedolizumab therapy in IBD. The data were also reported in the Main Text, as Figure 3.

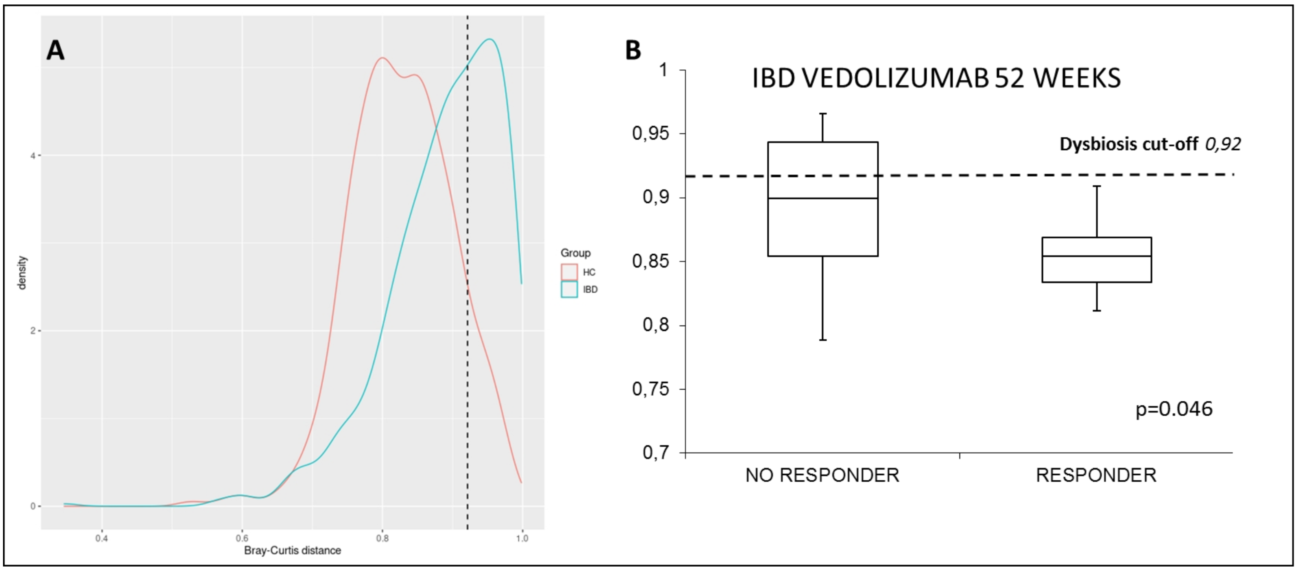

1.5.4 - Results of the Cross-correlation Analysis

Microbial networks were constructed by calculating pairwise Pearson correlation coefficients of microbial abundances to quantify 'co-occurrence' across all study groups, namely, individuals with HC, IBD, UC and CD. The figure is also reported in the Main Text, as Figure 4.

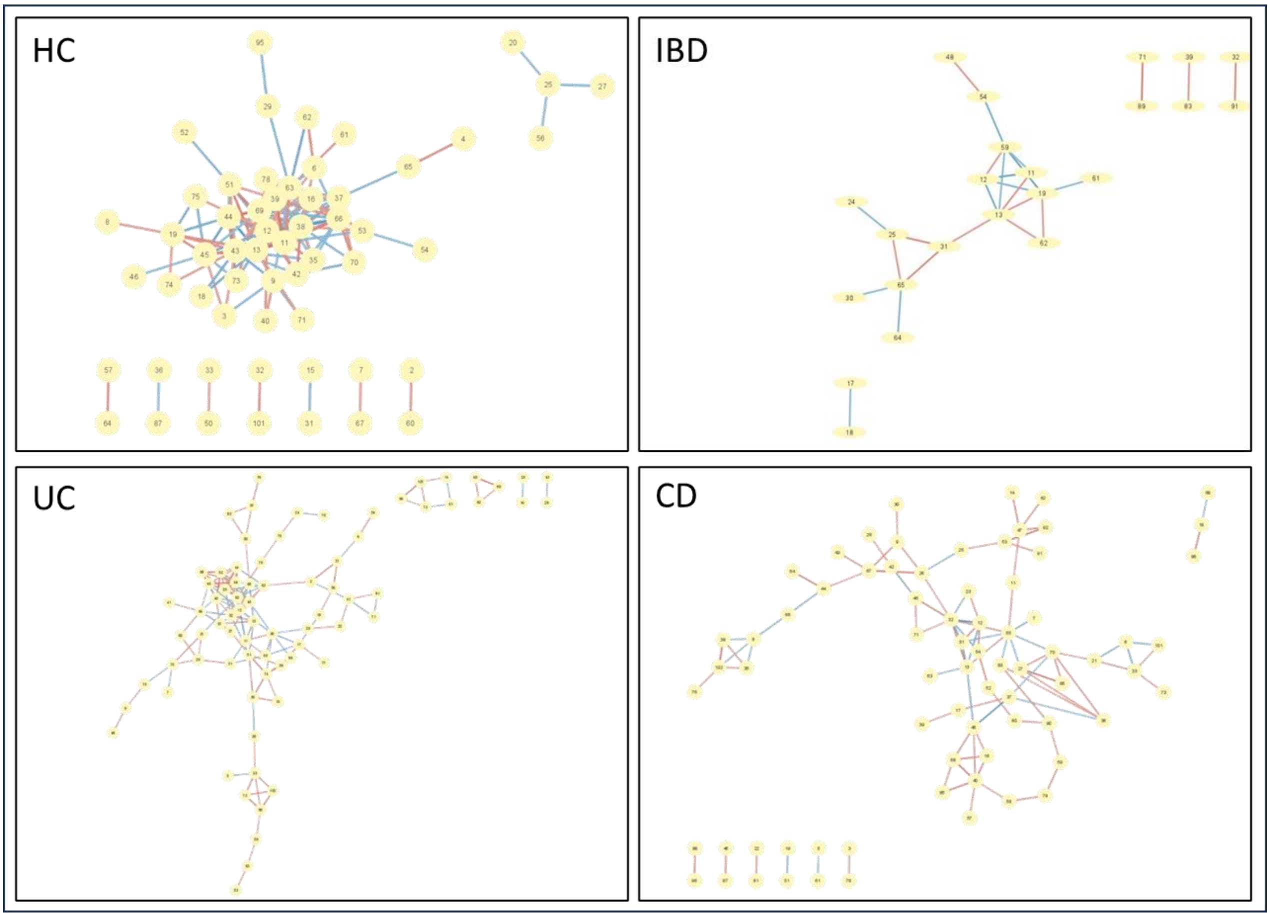

Supplement: Supplementary file 1 [file microorganisms-12-01260-s001.zip › Supplementary_File_S1.pdf]
